# Prediction of Female Breast Cancer Incidence among the Aging Society in Kanagawa, Japan

**DOI:** 10.1371/journal.pone.0159913

**Published:** 2016-08-17

**Authors:** Kayoko Katayama, Hiroto Narimatsu

**Affiliations:** Cancer Prevention and Control Division, Kanagawa Cancer Center Research Institute, Yokohama, Kanagawa, Japan; Taipei Medical University, TAIWAN

## Abstract

Owing to the increasing number of elderly “baby boomers” in Japan, the number of cancer patients is also expected to increase. Approximately 2 million baby boomers from nearby local areas are residing in metropolitan areas; hence, the geographical distribution of cancer patients will probably markedly change. We assessed the expected number of breast cancer (BC) patients in different regions (urban, outer city, town, rural) using estimates of the nation’s population and Kanagawa Cancer Registry data. To estimate future BC incidence for each region, we multiplied the 2010 rate by the predicted female population for each region according to age group. The incidence cases of BC in those aged ≥65 years is expected to increase in all areas; in particular, compared to rates in 2010, the BC incidence in urban areas was predicted to increase by 82.6% in 2035 and 102.2% in 2040. Although the incidence in all BC cases in urban areas showed an increasing trend, until peaking in 2040 (increasing 31.2% from 2010), the number of BC patients would continue to decrease in other areas. The number of BC patients per capita BC specialist was 64.3 patients in 2010; this value would increase from 59.3 in 2010 to 77.7 in 2040 in urban areas, but would decrease in other areas. Our findings suggest that the number of elderly BC patients is expected to increase rapidly in urban areas and that the demand for BC treatment would increase in the elderly population in urban areas.

## Introduction

In 2014, the proportion of elderly citizens in the Japanese population was 26.0% [1]. Such an ultra-aged society has not been observed in any other country [2]. One particular age group that has had a marked influence on this rapid demographic change is the “baby boomer generation,” which includes individuals born during the first baby boom (1947–1949) after World War II. This group has been a dominant component of the Japanese population, and the total number of births is 8,060,000 [3]. Approximately 2 million baby boomers moved from their hometown to major metropolitan areas, such as the Tokyo metropolitan area, for higher education or employment [4]. Based on data from the Ministry of Health, Labour and Welfare, it appears that these individuals tend to remain in these areas after their retirement [5, 6]. Hence, it is considered that the number of elderly people in urban areas will rapidly increase in the near future in Japan, and hence, communities in urban areas will age. Accordingly, it is reasonable to predict that the demand for medical care for cancer will also increase in urban areas as the number of elderly cancer patients increases.

Medical care for cancer requires vast medical resources. Hence, cancer is an important disease for health care policy and clinical medicine. Many countries have initiated measures against cancer, such as anti-smoking measures and promotion of cancer screening. The estimation of future cancer mortality and cancer incidence has played a major role in the drafting and evaluation of these national measures [7–9].

The geographic distribution of cancer patients will also markedly change; in particular, the number of patients in urban areas will show a disproportionate increase. Hence, appropriate regional measures will need to be taken, while considering the demographic movement in each region, and measures at the national level will also be necessary. Similar situations are noted in other developed countries, wherein rapidly aging communities in large cities are increasing as a result of industrialization [10]. To implement regional anti-cancer measures, regional statistics as well as national statistics on cancer incidence and cancer-related mortality are needed. However, the accumulated evidence from regional studies is currently insufficient.

In the present study, we assessed the future breast cancer (BC) incidence in Kanagawa Prefecture, which is adjacent to Tokyo and has the largest population in Japan (approximately nine million) (S1 Fig). To predict the changes in medical care demands associated with societal aging in each medical region, we assessed the future regional number of BC patients using existing data of the estimated future population and the Population-based Cancer Registry data. BC is the most common cancer among women in Japan. Moreover, the incidence of BC is predicted to increase in the near future owing to societal aging in developed countries; projections based on the GLOBOCAN 2012 predict a substantive increase to 19.3 million new cancer cases per year by 2025, due to growth and aging of the global population [11]. In recent years, the age-specific mortality and incidence rates of BC in the elderly has continued to increase in Japan. The age-specific mortality rate for people aged ≤65 years has increased from 31.7 per 100,000 in 2005 to 36.4 per 100,000 in 2009; the incidence rate for the same population increased from 108.2 to 138.5 per 100,000 in the same period [12] (S2 Fig). Measures against BC such as BC screening are likely to have an impact on medical care policy, such as promotion of cancer screening and providing universal medical care that ensures sufficient quality and quantity of care for cancer patients. These measures are re-evaluated every 5 years.

The Population-based Cancer Registry system of Kanagawa Prefecture has useful data that could enable an accurate estimation. Moreover, Kanagawa Prefecture includes Yokohama City, with a population of ≥3.7 million, as well as mountain villages in the western region. Thus, the region includes urban and other areas, and can serve as a good model for simulating community aging in urban areas nationally. We believe that this study provides important evidence for establishing optimal anti-cancer measures for an aging society, which is a common challenge in developed countries.

## Materials and Methods

### Data collection

**Fig 1:**
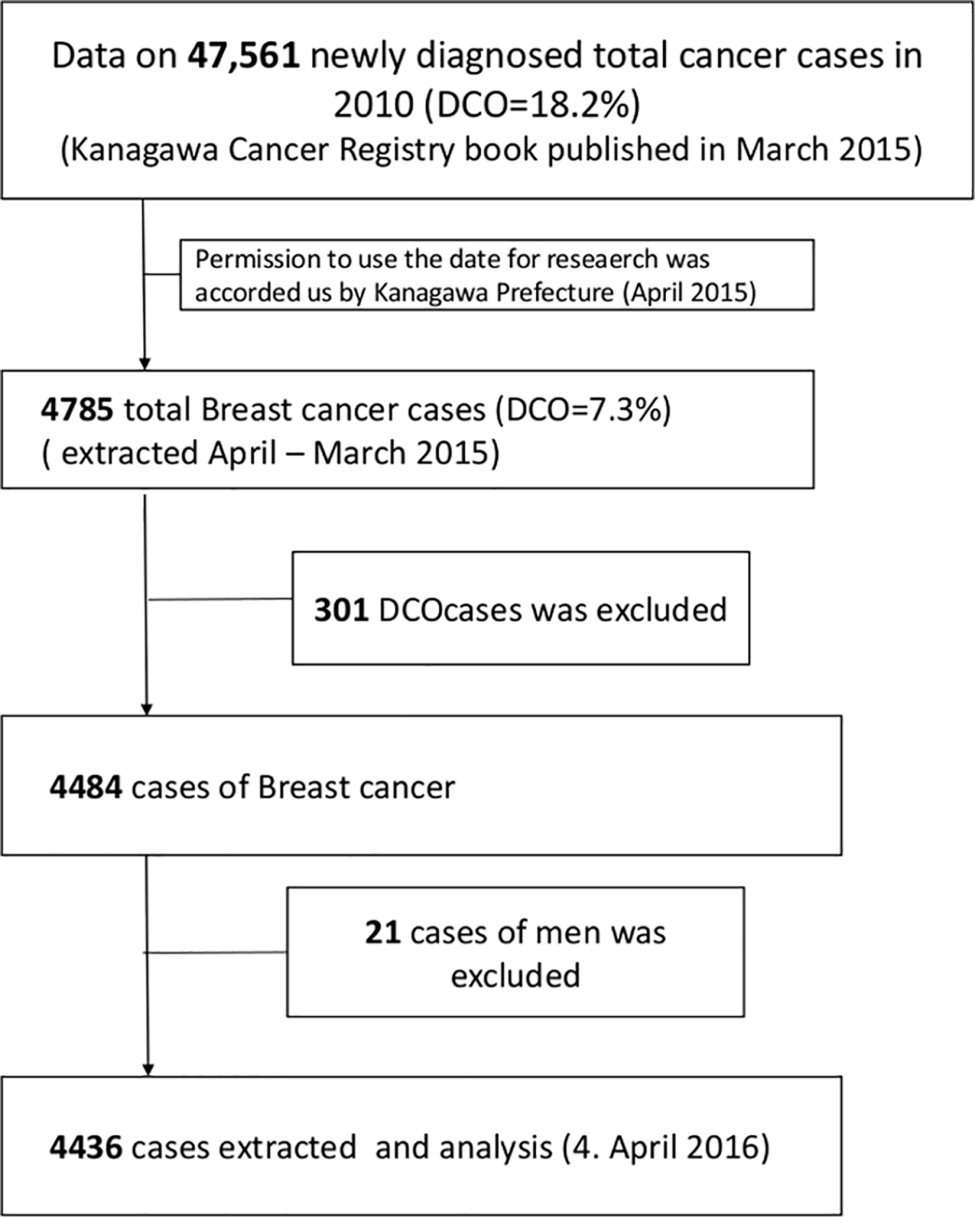
A Flow Diagram of the Data Collection Methods.

In 2010, 45 prefectures in Japan had population-based cancer registries; the two prefectures that had not developed these registries were the Tokyo and Miyazaki Prefecture. Twelve of the registries were from three metropolitan areas, Capital Tokyo, Kinki, and Chukyo. The remaining 33 prefectures with registries were local areas, including 32 prefectures and Hiroshima-city [13]. The cancer registry in Kanagawa Prefecture—initiated in 1970—is one of the population-based regional cancer registries in Japan. In 2010, the population of Kanagawa Prefecture was 9,048,331, and only Tokyo had a larger population. Data on 4,436 newly diagnosed BC cases were provided by the Kanagawa Population-based Cancer Registry from a total of 46,966 cancer cases in 2010, after obtaining permission from Kanagawa Prefecture in April 2015 [14]. The cases identified using death certificate only (DCO) were excluded because detailed information including the cancer diagnosis date and incidence information were not obtained for those cases (Fig 1). BC cases identified using DCO comprised 7.3% of all cases in 2010[14]. The Population-based Cancer Registry provides data regarding initial diagnosis date, history of discovery, classification of malignant tumors, stage and treatment. However, in this study, we did not use these data; instead, we used incidence year, age at diagnosis, and address. Subjects were divided into four age groups: 0–14 years, 15–39 years, 40–64 years, and ≥65 years. The reason that we used these age groups was owing to the age distribution of the BC patients. In Japan, 40 years of age is the key age for BC, as the age-specific BC incidence rate rapidly increases at age ≥40 years. Moreover, BC screening is commonly indicated for patients aged 40 years in Japan. Another key age is 65 years, as the therapeutic strategy usually differs between patients aged ≥ 65 years and those aged <65 years. Those patients aged <65 years usually can undergo intensive treatment, including curative operation and chemotherapy. As an indicator of the health care demands for BC, we assessed the estimated incidence index value of BC.

Data on 67,478 newly diagnosed BC cases from 1991 to 2010 for prediction using the Nordpred package [15], were also provided by the Kanagawa Population-based Cancer Registry, after obtaining permission from Kanagawa Prefecture in April 2016.

**Fig 2:**
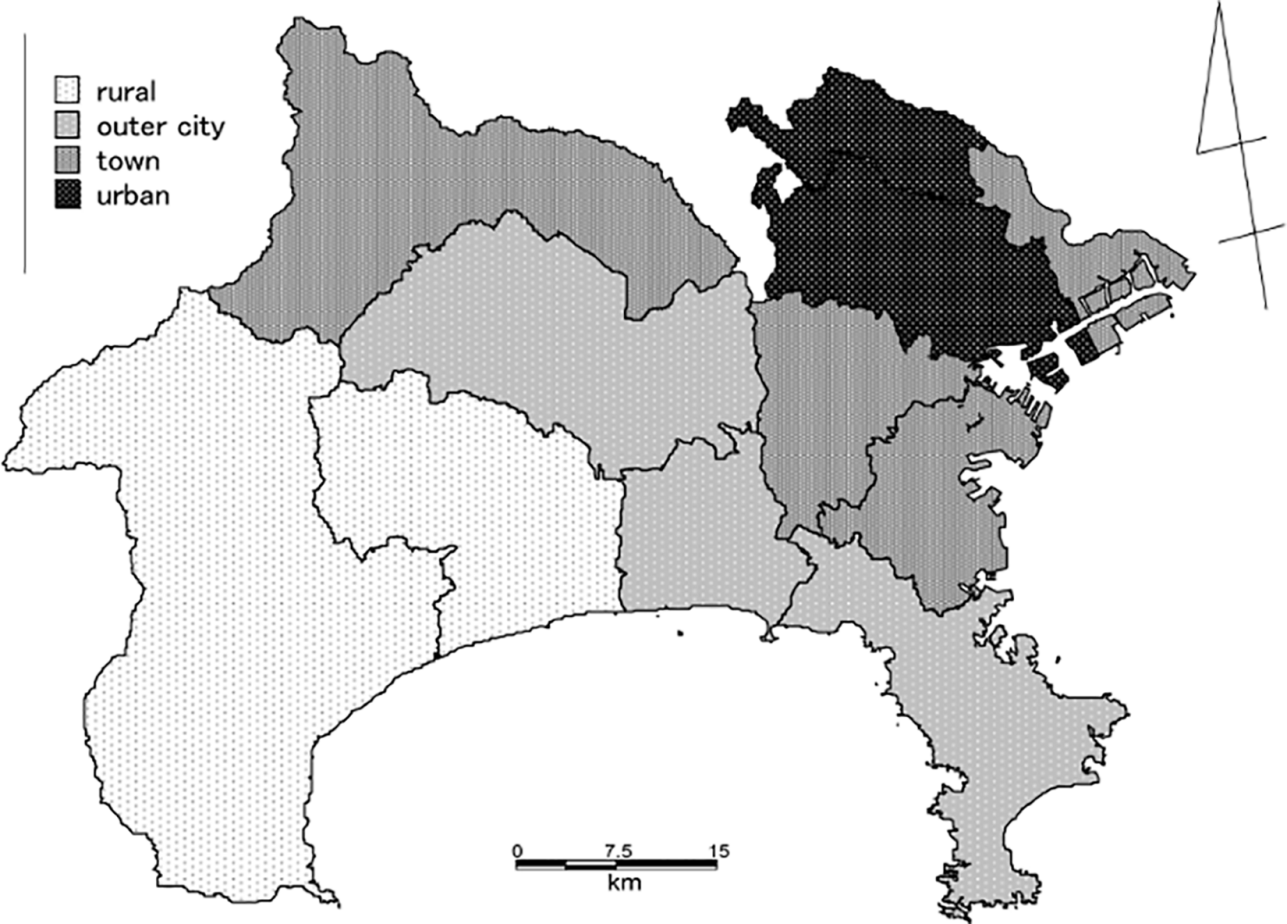
Kanagawa Prefecture Divided into Four Group Areas. Kanagawa Prefecture can be classified into 4 categories (rural, outer city, town and urban areas) depending on geographical features (Table 1). The geographical information system (GIS) data of the map was used under a CC BY license, with permission from Mitsui Zosen Systems Research Inc., original copyright 2010.

To establish a regional medical treatment plan, certain “medical regions” are determined by the Japanese government, according to the Medical Service Act. There are three types of medical regions based on the geographical connections. Accordingly, a prefecture can be divided into 3–20 regions: primary (equal to municipality), secondary, and tertiary (equal to prefecture). A flow chart explaining the collection of regional data is shown in Fig 1. There are 11 secondary medical regions in Kanagawa (Table 1, S3 Fig). As shown in Table 1, we classified these 11 regions into four groups based on the geographical features and population density: urban, town, outer city, and rural areas (Fig 2, S1 and S2 Tables). The urban areas included two regions: Yokohama North (the most populous region, with many large-scale commercial facilities and high land values) and Kawasaki North (the region with the highest population density, with good transportation access as it is adjacent to the Tokyo metropolitan area). The town areas included four regions: Yokohama West, Yokohama South (the second most populous region, with an increasing population since the 1950s, also known as the “bad town” of Yokohama-city from the 1950s; this region has shown a high aging rate in recent years), Kawasaki South (the region with the second highest population density and an industrial area), and Sagamihara (widely includes urban areas, but also includes some local areas). The outer city areas included three regions: Yokosuka-Miura, Shonan East, and Central (adjacent to Yokohama city). The rural areas included two regions: Shonan West (small population) and Western Kanagawa (mountainous terrain); both rural areas have a high rate of aging (Table 1, S4 Fig).

**Table 1:** Geographical Features of Secondary Medical Regions.

Area name	Classification of the area	Ground area (km^2^)	Population(2010)	Density of population	Number of BC^a^ incidence(2010)	Number of BC specialist^b^	Number of Patiennts per capita BC specialist (2010)	Feature of area
Yokohama North	Urban center	177.1	1,518,277	8,573.0	742	4	185.5	It is the one of Yokohama-city. This area is so good transportation access that it is adjacent to Tokyo metropolitan. This area has many large-scale commercial facilities, and high land values.
Yokohama West	Town	138.2	1,109,522	8,028.4	594	13	45.7	The population in this area increased as bed town of Yokohama-city from the 1950s, but an aging rate is high in recent years.
Yokohama South	Town	122.1	1,060,974	8,690.8	603	10	60.3	This area is sea side. The population in this area increased as bed town of Yokohama-city from the 1950s, but which has a high aging rate in recent years.
Kawasaki North	Urban center	78.7	820,047	10,414.6	384	15	25.6	This area is so good transportation access that it is adjacent to Tokyo metropolitan. Progress in rapid population growth and urbanization as Tokyo bed Town from 1960s.
Kawasaki South	Town	64.0	605,465	9,466.3	211	2	105.5	This area is seafront, there has been an industrial area, including large-scale petrochemical complex.
Yokosuka・ Miura	Outer city	207.0	732,059	3,536.7	358	6	59.7	This area includes the Miura Peninsula, and facing the Tokyo Bay and Sagami Bay. Once this area has been developed as bed Town of Tokyo and Yokohama, followed by a subsequent population decline in 1992, there is a high rate of aging.
Shonan East	Outer city	118.6	692,410	5,836.2	231	1	231.0	This area is facing the Pacific Ocean. The current, this area is bed town of Tokyo and Yokohama- city.
Shonan West	Rural area	253.2	594,518	2,347.8	278	6	46.3	Here is the foot of the Tanzawa Mountains. On the other hand, this area is rice and dairy farming thriving region.
Central	Outer city	292.8	792,916	2,708.0	477	3	159.0	Located in the center of the prefecture. This area has good access to Tokyo, it is functioning as bed Town.
Sagamihara	Town	328.8	717,544	2,182.1	341	8	42.6	This area is adjacent to Tokyo, on the other hand, adjacent to the local area, Yamanashi prefecture. Population was increased by the merger of the town. In recent year, the large-scale factory withdrew have been an increasing number of roles as a commuter town.
West	Rural area	635.3	359,051	565.2	217	1	217.0	This area is a mountainous continuing to Hakone. There is also a spa town since ancient times.
Kanagawa Prefecture totoal		2415.9	9002783	62,349.1	4436	69	64.3	

^a^BC = Breast cancer

^b^The number of breast cancer specialists (Published The Japanese Breast Cancer Society in April 2015)

Using the 2010 census population data from the National Census and the estimated data for future population up to 2040 from the National Institute of Population and Social Security Research, we used these population data for each secondary medical region (S3 Table). The population prediction value was calculated using the cohort component method by the National Institute of Population and Social Security Research. [16]. The detailed method is described elsewhere. Briefly, the projections using the cohort component method require: 1) basic population, 2) future fertility rate, 3) future survivorship rate, 4) future net migration rate, and 5) future sex ratio at birth. In addition, after the populations of prefectures were projected by the cohort component method, the projections were adjusted for median sex and age [16].

As an indicator of the health care provision for BC, we extracted information on the number of BC specialists and the hospitals where they work in 2015, from the website of the Japanese Breast Cancer Society [17]. In brief, experienced medical doctors who completed the educational programs specified by the society are certificated as BC specialists. As of 2015, 69 doctors were certified as BC specialists in Kanagawa Prefecture.

### Data Analysis

#### Prediction using the rate in 2010

Future estimates of BC incidence were calculated from the 2010 incidence data from the cancer registry and were predicted using the nation’s population data. This estimation method for future BC incidence was based on the assumption that the rates for cancer onset would be the same in a specific age group [9]. To estimate future BC incidence according to the medical region, we calculated the estimated BC incidence by multiplying the incidence rate in 2010 by the estimated female population in each region according to age groups (0–14, 15–39, 40–64, and ≥65 years). For example, the predicted incidence in 2020 was calculated as follows:

$$\begin{array}{l} Predicted\;BC\;incidence\;in\;the\;group\;aged\;40–64\;years\;(/10^{5}people)\;in\;2020\\ \;=\{incidence\;rate\;in\;the\;group\;aged\;40–64\;years(/10^{5}people)in\;2010\}\\ \times \{the\;predicted\;population\;in\;the\;region\;aged\;40–64\;years\;in\;2020(/10^{5}people)\} \end{array}$$

The future estimated values were presented as an index value, and were expressed as a relative value in comparison with the 2010 incidence as a proportion of 100.

The distribution of BC specialists in each medical region was estimated using Tango’s test and Pearson’s chi-squared test using R version 3.2.5 (R Foundation for Statistical Computing, Vienna, Austria), including the packages “spdep”, “Dcluster”, and “classInt” [18, 19]. Of several methods for spatial clustering of hypothesis testing, we adopted Tango’s test [20] because it can analyze the presence of clustering considering geographic proximity of spatial objects. This statistical model may be better fitted to the spatial distribution of the patients.

The data used are shown in Table 1 (and S1 Table). The data included the names of the municipalities in Kanagawa (as names and regions); the x and y coordinates of the municipalities on a plane rectangular coordinate system; the longitudes and latitudes of the municipalities; the population; and the incidence of BC. The number of BC specialists in the municipalities was included as an objective variable, whereas the number of physicians in the municipalities was included as an explanatory variable.

Using MapInfo Professional, version 11.0 (Pitney Bowes Software K.K., Japan, http://www.mapinfo.jp/location/integration.html), we visualized the study design to predict the future incidence of BC by processing the following provided geographical information system (GIS) data. The GIS data of Kanagawa Prefecture and whole Japan were used with permission. The copyright of the data belongs to the Japan Map Center (Tokyo, Japan).

#### Prediction using the Nordpred package

We used a modified age-period-cohort (APC) model proposed by Moller et al., for comparison with the prediction simple model, in which the incidence rate in 2010 was used for the projection [21]. As described in a previous study [22], we conducted the future prediction using this modified APC model with the Nordpred package via the R software (http://www.kreftregisteret.no/software/nordpred). The Nordpred package derives the relevant parameters from past observations and uses them to estimate future incidence using the APC model. The Nordpred package employs the so-called power 5 link function instead of the long link function, as this was shown to improve the accuracy of the projections of the past trends [23, 24]. We fitted the model to the observed incidence starting from 1991 to 1995 according to 5-year periods and 5-year age groups and projected the linear time trend with adjustment for the period and cohort effect to predict the incidence for 2011–2015, 2016–2020, 2021–2025, 2026–2030, and 2031–2035. As an approximation, these 5-year periods were used to represent the years 2015, 2020, 2025, 2030, and 2035, respectively. The data for the population of the whole of Kanagawa during 1991–2010 and the forecasted population data in years 2015–2035 were used for the calculation. The single-year populations of the four regional areas in 1995, 2000, 2005, and 2010 was multiplied by five and these were used for the calculation of the population in 1991–1995, 1996–2000, 2001–2005, and 2006–2010, respectively. For the age-adjusted incidence of cancer, we used the world standard population (Doll and Smith) for age standardization. The Nordpred package for R that we used for the prediction is available via the website of the Cancer Registry of Norway [15]. All analyses were carried out with R statistical software version 3.2.5; in particular, the R package “Nordpred” was used for fitting the APC models.

## Results

### Estimation of future BC incidence

#### Prediction using the rate in 2010

**Fig 3:**
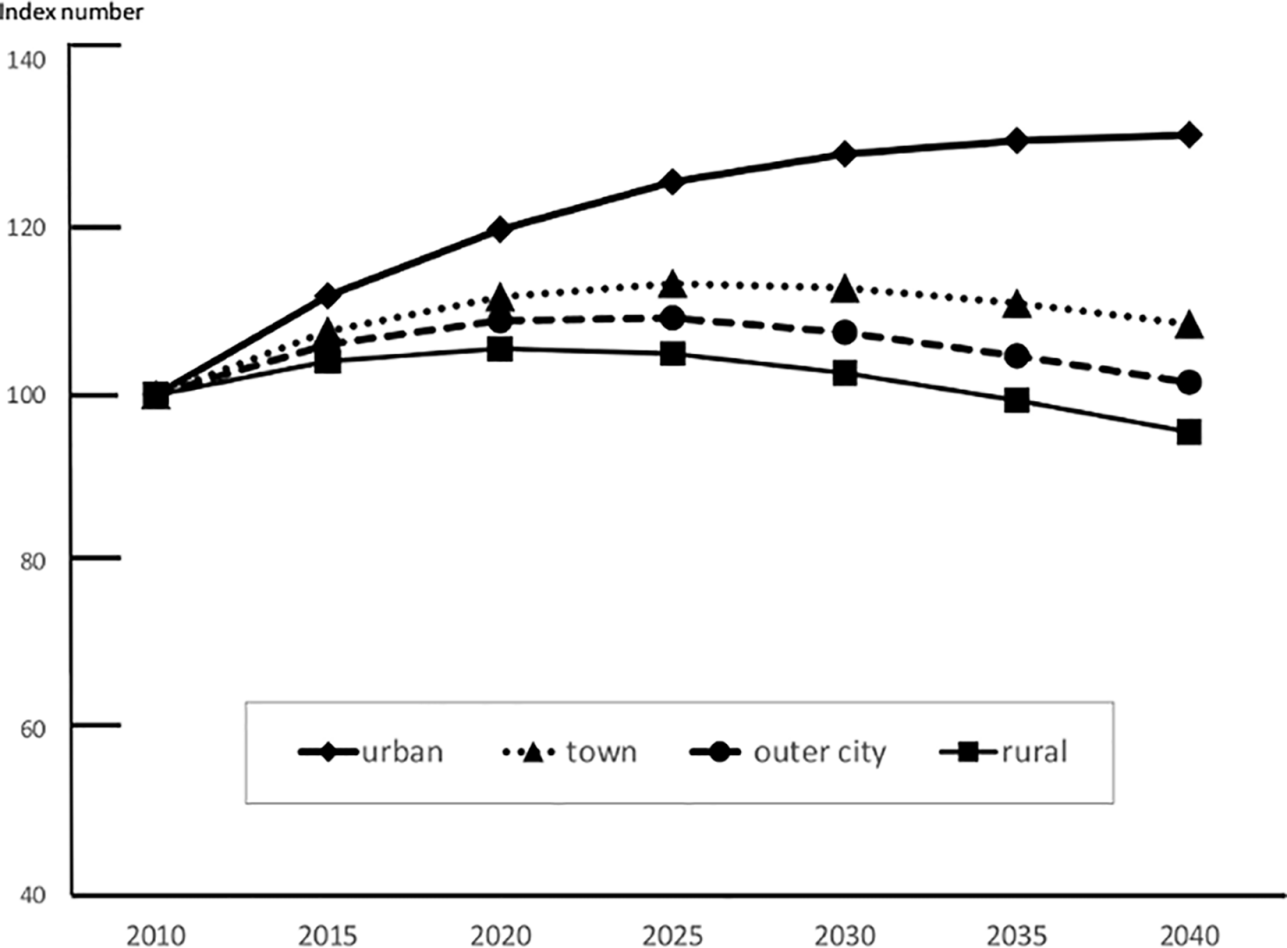
Estimation of Future Breast Cancer Incidence in Kanagawa for Each Area (All Age Groups). The future estimated values are presented as an index number, and are expressed as relative value in comparison with the 2010 incidence as a proportion of 2010 for four areas.

**Fig 4:**
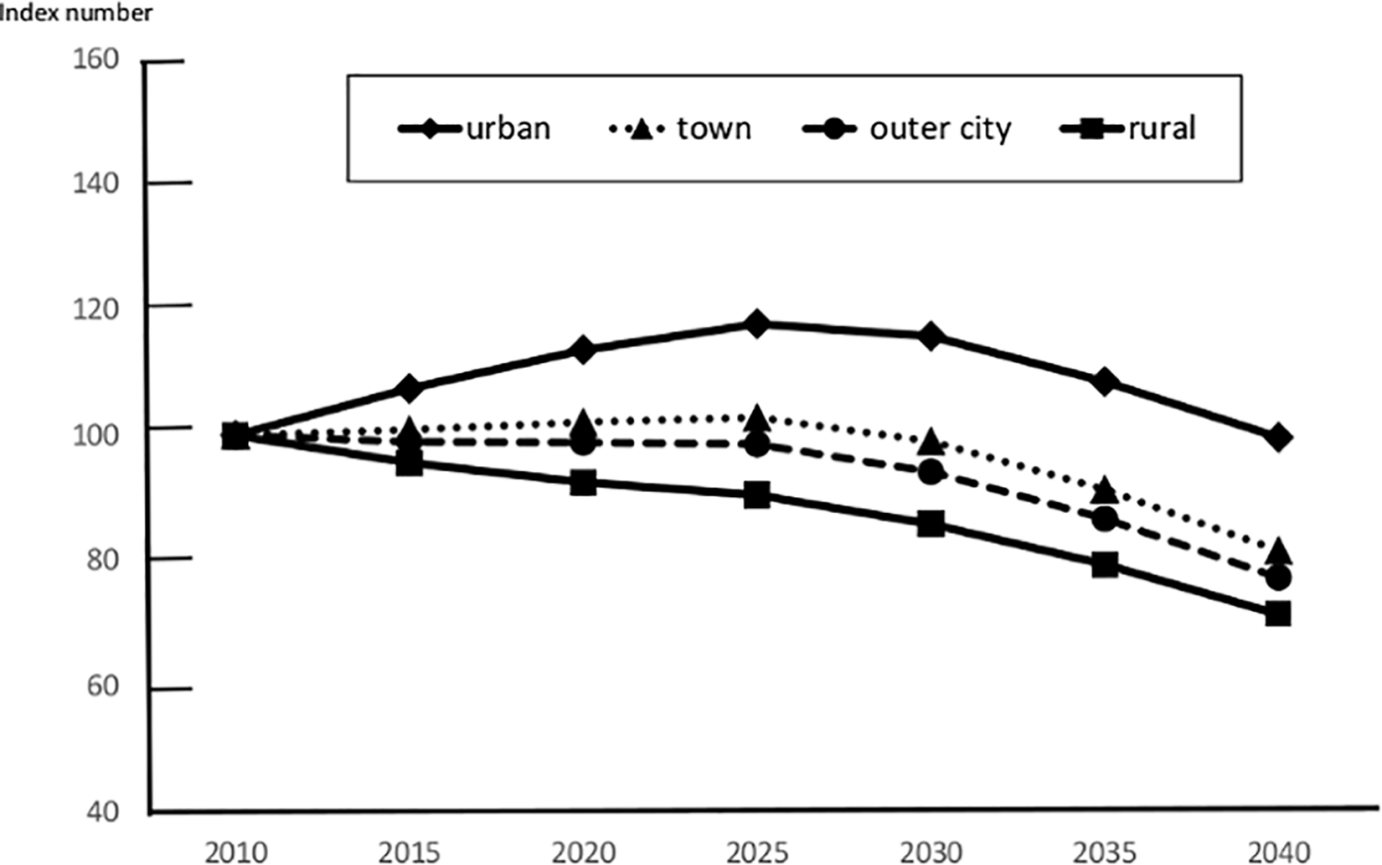
Estimation of Future Breast Cancer Incidence in Kanagawa for Each Area (40–64 Years Old). The future estimated BC incidence values for those aged 40–64 years are presented as an index number, and are expressed as relative values in comparison with the 2010 incidence as a proportion of 2010 for four areas.

**Fig 5:**
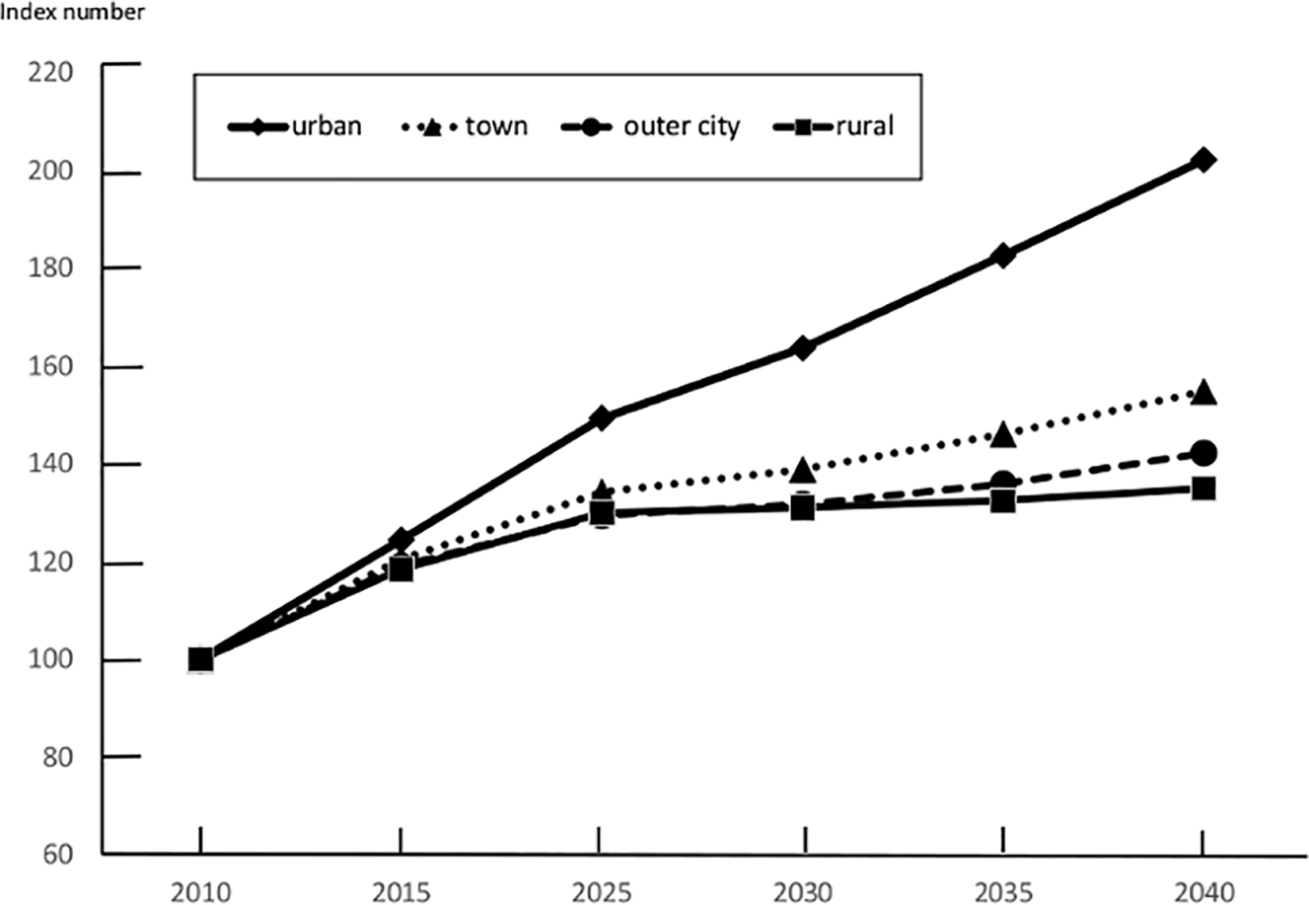
Estimation of Future Breast Cancer Incidence in Kanagawa for Each Area (≥65 years). The future estimated BC incidence values for those aged ≥65 years are presented as an index number, and are expressed as relative values in comparison with the 2010 incidence as a proportion of 2010 for four areas.

The number of BC patients in each secondary medical region in 2010 is shown in Table 1. The urban areas showed a tendency to increase to a peak value in 2040 (increasing by 31.2% from 2010); the incidence in other areas showed a decreasing trend from 2035. However, the decreasing trend was highest in the rural areas (decreasing by 4.4% in 2040) (Fig 3). The estimated incidence by age group is shown in Figs 4 and 5. The incidence in the 0–14-year age group in 2010 was 0 in all areas. Therefore, BC incidence in this age group was predicted to decrease, without an attendant increase, depending on population estimates in all areas. We also predicted that BC incidence in the 15–39-year age group would decrease in all areas, with the largest decrease of 40.6% decrease in the west (rural area) in 2040. The BC incidence in individuals aged 40–64 years is expected to increase in urban and town areas after peaking in 2025, but is expected to decrease slowly after 2030. It was predicted that BC incidence in outer and rural areas would continue to decrease after 2010 (Fig 4). The BC incidence in individuals aged ≥65 years is expected to increase continuously in all areas; among these, the rate of increase was predicted to be highest in the urban areas (82.6% increase in 2035 and 102.2% increase in 2040 as compared to that in 2010; Fig 5).

#### Comparison with the prediction using the Nordpred package

We summarized the results of the predictions using the Nordpred package in Table 2 and provide a comparison with those using the rate in 2010. With the Nordpred package the total incidence of BC in the whole of Kanagawa would increase from 4,436 in 2010 to 8,979 in 2035, which was more than 1.8 times higher than the prediction using the rate of 2010: 5,018 cases /year. In the four regions studied (urban, town, outer city, and rural), the predicted incidences of BC would be higher than those using the rate in 2010 in all periods (2015–2035; Table 2, S5 Fig).

**Table 2:** Estimation of breast cancer incidence cases (/year) and age standardized rates.

Year	Areas	Incidnces cases (/year)		Age-standardized rates^a^ (/100.000)	
Obseved data (Data from the cancer registry)					
2010	Whole Kanagawa^c^	4,436		60.4	
	Urban area	1,126		NA	
	Town area	1,749		NA	
	Outer city	1,066		NA	
	Rural area	495		NA	
Future Prediction					
		Nordpred	Prediction using the rate in 2010^b^	Nordpred	Age-standardized rate of 2010
2015	Whole Kanagawa^c^	5,611	4,789	67.0	60.4
	Urban area	1,994	1,260	97.1	NA
	Town area	2,261	1,883	73.9	NA
	Outer city	1,836	1,131	85.2	NA
	Rural area	740	515	84.5	NA
2020	Whole Kanagawa	7,014	4,987	78.8	60.4
	Urban area	2,930	1,349	130.1	NA
	Town area	2,803	1,954	85.6	NA
	Outer city	2,267	1,161	99.6	NA
	Rural area	996	523	111.6	NA
2025	Whole Kanagawa	8,048	5,080	86.3	60.4
	Urban area	3,725	1,413	153.2	NA
	Town area	3,141	1,983	92.2	NA
	Outer city	2,510	1,164	107.3	NA
	Rural area	1,203	520	133.1	NA
2030	Whole Kanagawa	8,553	5,078	88.7	60.4
	Urban area	4,142	1,451	159.9	NA
	Town area	3,250	1,973	91.2	NA
	Outer city	2,472	1,146	107.9	NA
	Rural area	1,303	508	145.8	NA
2035	Whole Kanagawa	8,979	5,018	91.3	60.4
	Urban area	4,641	1,469	167.9	NA
	Town area	3,304	1,941	90.7	NA
	Outer city	2,497	1,116	110.6	NA
	Rural area	1,398	492	160.6	NA

NA: indicates not applicable.

a: According to the world standard population (Doll and Smith)

b: We calculated the estimated BC incidence by multiplying the incidence rate in 2010 by the estimated future female population.
$$\begin{array}{l} Predicted\;BC\;incidence\;in\;the\;group\;aged\;40–64\;years\;(/10^{5}people)\;in\;2020\\ \;=\;\{incidence\;rate\;in\;the\;group\;aged\;40–64\;years(/10^{5}people)in\;2010\}\\ \times \{the\;predicted\;population\;in\;the\;region\;aged\;40–64\;years\;in\;2020(/10^{5}people)\} \end{array}$$

c: Including 4 areas.

### Balance of supply and demand of medical care for breast cancer

**Fig 6:**
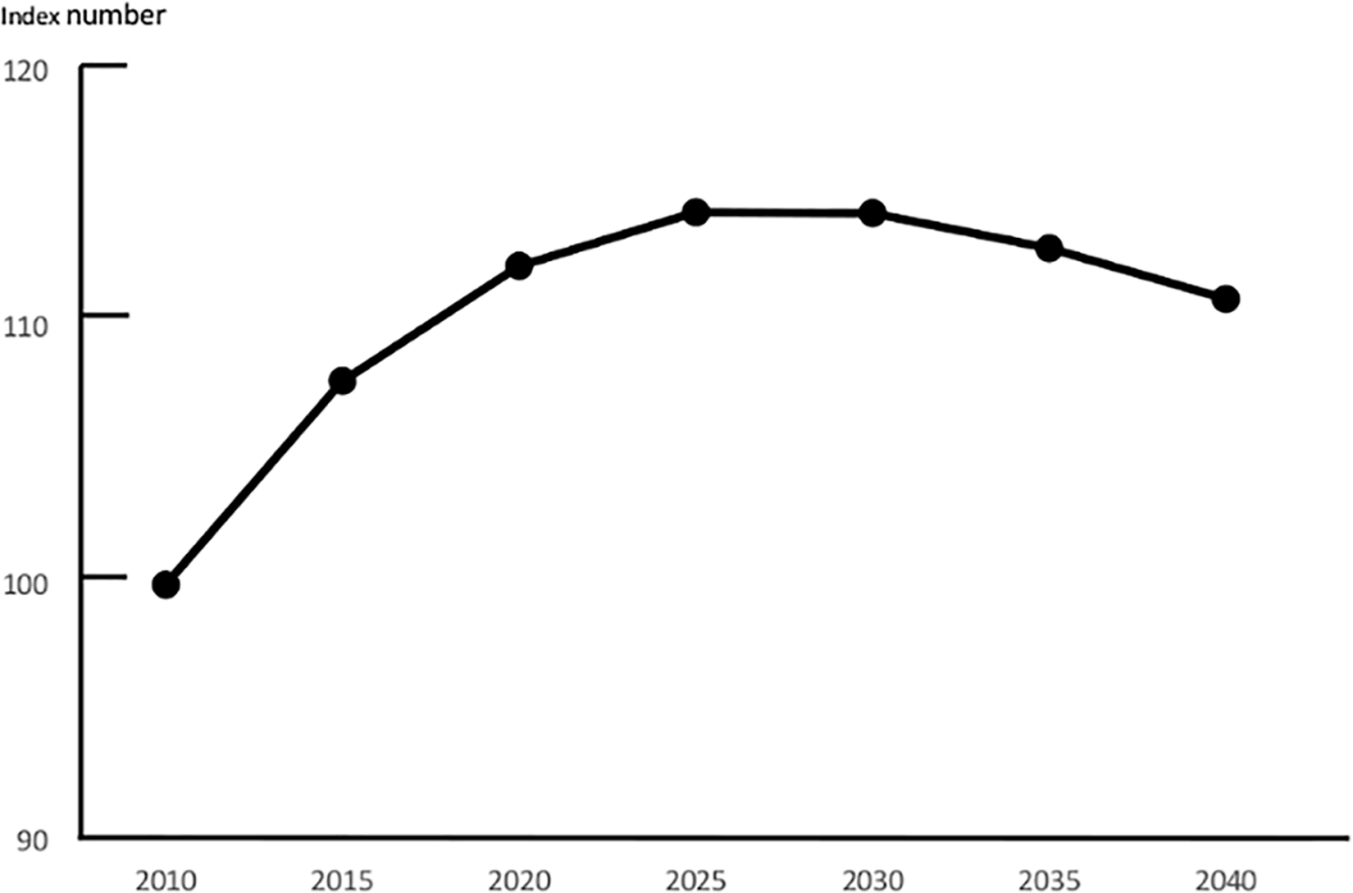
The Predicted Total Number of Patients with Breast Cancer in Kanagawa as a Whole. The future estimated numbers of patients of all ages with breast cancer are presented as an index number and are expressed as relative values in comparison with the 2010 incidence as a proportion of 2010.

**Fig 7:**
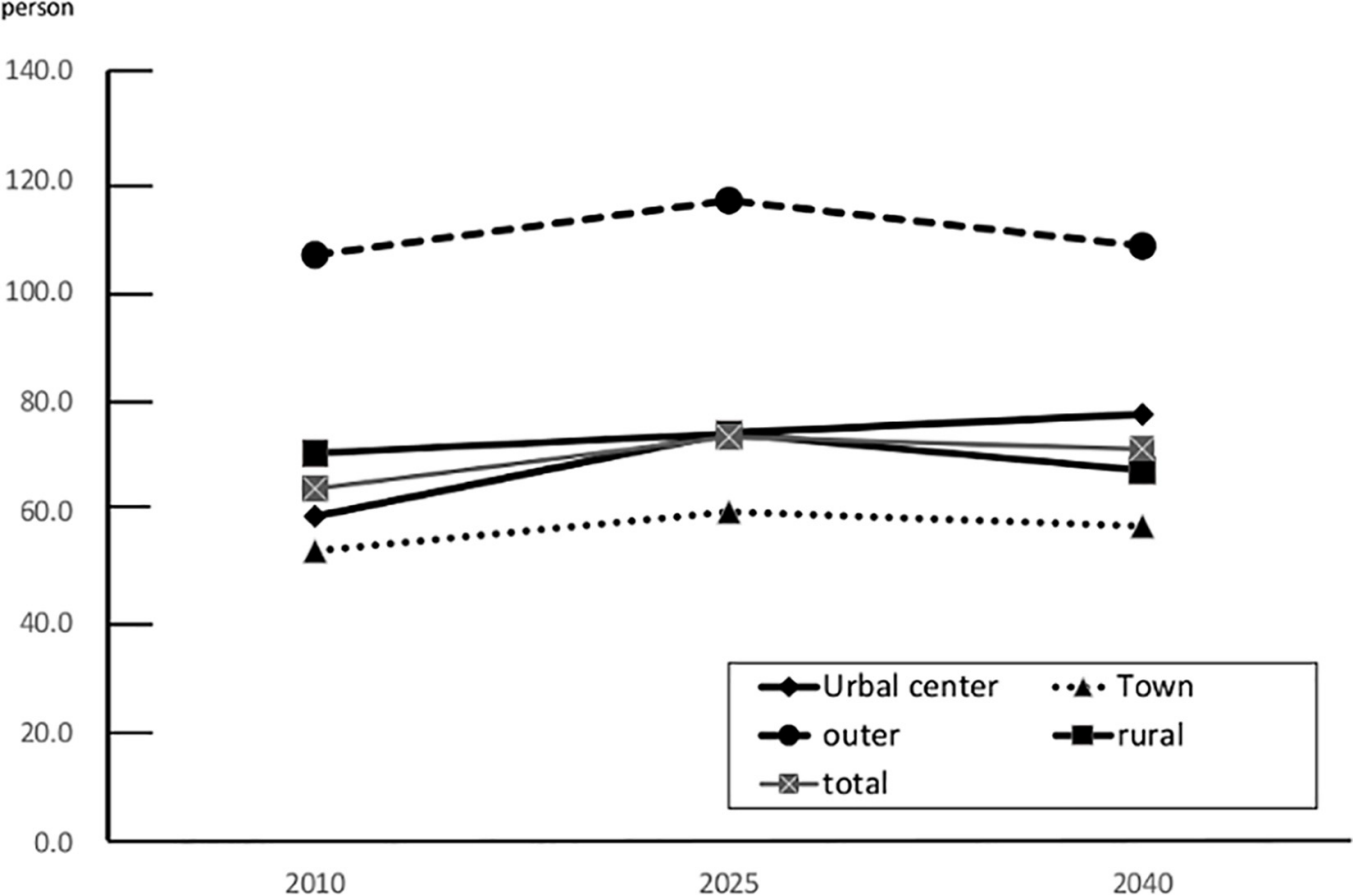
The Number of Patients per Capita Breast Cancer Specialist for Each Area.

The total number of new BC patients in Kanagawa in 2010 was 4,436, which was predicted to increase to 5,080 in 2025 and to decrease to 5018 in 2035 (Fig 6). Table 1 shows the present distribution of BC specialists in Kanagawa Prefecture. BC specialists were significantly unevenly distributed (Pearson: p = 0.008; Tango: p = 0.009). The number of BC specialists was greatest in urban areas (15 in Kawasaki North area), and was lowest in rural areas (one in the Kawasaki West area) and in an outer city area (one in the Shonan East area). In Kanagawa Prefecture, the number of patients per capita BC specialist was 64.3 in 2010. Based on the assumption that the distribution of BC specialists will remain constant, this value was expected to change to 73.6 patients in 2025 and 71.4 patients in 2040 (Fig 7). This value would further increase from 59.3 in 2010 to 77.7 in 2040 in the urban areas, but would decrease in the rural areas.

## Discussion

In the present study, we predicted that the demand for BC treatment would increase rapidly in the elderly population in the urban areas of Kanagawa Prefecture. The main reasons for this increase may be the large number of baby boomer residents in urban areas, who would be aging in the near future. For example, the population of elderly citizens (≥65 years of age) in urban areas is expected to reach 359,769 by 2030, which is 2.3 times higher than the estimated corresponding population of 156,446 in mountain villages (S4 Table). The housing policy of the government, which encouraged baby boomers to reside in urban areas, had a considerable influence on this polarized distribution. In addition, people in these areas commute to the Tokyo metropolitan area—a large center of commerce and industry in Japan. Thus, the establishment of good public transportation systems and the support of housing support policy led to the increased influx of a large number of baby boomers in urban areas from other local regions [5, 25].

The crude BC incidence rate rapidly increases at 45–50 years of age, and remains high after 50 years of age [26]. Hence, sharper increases in the number of BC patients are expected in urban areas rather than in rural areas. As baby boomers populate the big metropolitan areas located close to centers of commerce and industry [27], similar increases in the number of BC patients in the three so-called metropolitan areas of Kanto, Kansai, and Nagoya are inevitable.

This study showed that the balance between supply (number of BC specialists) and demand (number of BC patients) for medical treatment shifts with changes in the demand for BC treatment.

We predicted that the demographic distribution of regional cancer patients changed greatly, because the baby boomer generation living in Kanagawa tended to age concurrently. A comparison of the number of patients for every BC specialist among the four areas showed a two-fold increase between the area with the highest value (outer city areas) and the area with the lowest value (the town areas), clearly indicating an uneven distribution of BC specialists. This may be due to the lack of a university hospital with a medical faculty or their affiliated hospitals, which are the biggest sources of BC specialists in the outer city area, unlike the other three areas. If the current distribution of BC specialists persists, the supply-demand imbalance would worsen in urban areas (especially Yokohama North), whereas a better balance would be achieved in the other three areas. Therefore, the supply of medical care (the number of BC specialists) needs to increase in urban areas in the future.

The balance was changed when we hypothesized that the number of specialists under the present conditions would not change; however, we suggested that potential imbalances could be solved by moving BC specialists to high areas of demand. Another characteristic of urban areas is the predicted marked increase in the number of elderly patients, compared with that in other areas—the prediction showed that the number of BC patients in 2040 would more than double that in 2010. Elderly BC patients often have multiple underlying diseases and are likely to develop complications due to the cancer treatment [28], suggesting that they would require a vast amount of medical resources. In contrast, a sharper decrease in the total population is expected in rural areas compared to that in urban areas; thus, theoretically, an improvement in the supply-demand balance of medical care is predicted in rural areas. A decrease in patient density from 0.51 patient/km^2^ to 0.43 patient/km^2^ in rural areas, which is in sharp contrast to the increase in patient density from 4.33 patient/km^2^ to 4.71 patient/km^2^ in urban areas, would require the initiation of specific measures, as access to medical institutes in depopulated rural areas is expected to deteriorate. Hence, the establishment of public transport services (such as a community bus service) and remote medical care services using information and communication technology is required. Such a situation involving an increasing demand for BC treatment is probably common across Japan in other urban areas with a large number of baby boomers.

The present study predicts that needs in medical care will change in the future and that the strategy to treat BC would have to be individualized according to the number of patients, age distributions, and geographic characteristics of each region. This has a great impact on policies concerning cancer control, thus requiring a paradigm shift; this is especially true because the main goal of nationwide cancer control had been focused on providing universal access to cancer care and prevention in Japan, thus eliminating cancer-care disparities. This situation is inconsistent with that in England, where the future burden of cancers was predicted to be different between London and England as a whole [24]. Consistent with the findings of the current study, this prior study in England concluded that health care burdens are sensitive to demographic population trends. Therefore, future studies should investigate future cancer burdens and demographic population trends in other developed countries; this would provide an important framework for cancer policy in those countries.

**Fig 8:**
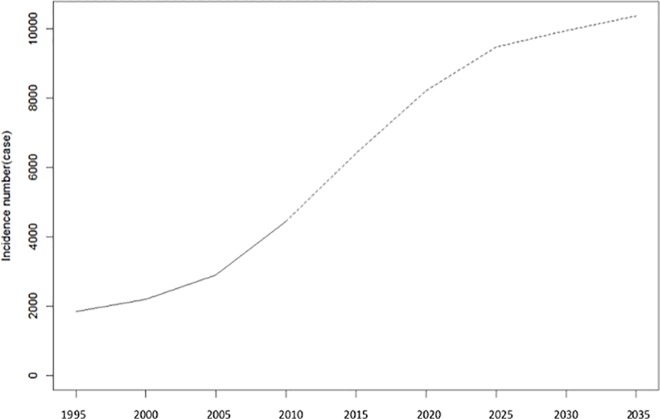
Trends and Projections of the Breast Cancer Incidence Cases (/year) using Nordpred in Kanagawa (All Age Groups). Incidence cases projections for all age groups using the projection from the Nordpred package.

This study provides important information that could help guide future cancer care policies. However, several points need to be addressed. First, to estimate the future incidence according to each medical region, we multiplied the incidence rate of 2010 by the population in the secondary medical regions. This estimation method, using the incidence data of a single year, can be applied to other municipalities (as our calculation did not consider the historical data of cancer incidence); however, unlike other methods, the influence of the cohort effect cannot be considered. As per the epidemiologic definition, a cohort effect occurs when different distributions of disease arise from a changing or new environmental cause that affects age groups in a different manner. Thus, a cohort effect is conceptualized as a period effect that is differentially experienced through age-specific exposure or susceptibility to that event or cause (i.e., interaction or effect modification) [29]. Thus, only the effect of aging was considered in this study. Smoking and obesity are known risk factors for BC [30, 31], and differences in these two factors among different cohorts may influence the estimations. However, their overall influence is predicted to be small, as investigations of the association between smoking and BC onset in previous cohort studies in Japan have yielded controversial results [32–34]. Differences in the prevalence of obesity among different cohorts might have only marginal effects on the prediction. A previous cohort study from Japan reported that higher BMIs were significantly associated with an increased relative risk of BC among pre-and postmenopausal women at baseline. However, the hazard ratio per 1 kg/m^2^ of BMI increase was rather small: 1.03 in premenopausal and 1.06 in postmenopausal women [31]. Second, prediction the APC model has been reported to be useful [35]. In the present study, we also predicted future incidence using this model with the Nordpred package [15, 21]. In contrast to our simple prediction model, which only considered an age effect in which the incidence rate would gradually decrease from 2020 for Kanagawa Prefecture as a whole and each of the four areas, the prediction using the Nordpred package showed a continuous increase from 2010 (Fig 8, S5 Fig, Table 2). Careful interpretation of this disparity is required, as the period and cohort effect were probably overestimated in the Nordpred prediction; the predicted incidence in 2035 using the Nordpred package is more than 1.8 times higher than that using the rate in 2010 (Table 2). In the Kanagawa Population-based Cancer Registry, the number of registered cancer cases had been increasing owing to the recently available more complete data; the DCO of the registry in 1990 was 33%, which improved to 18.2% in 2010. This improvement contributed to the recent increase in BC incidence in Kanagawa Prefecture: this probably caused the overestimation.

In Japan, medical instructions state that legally, all cancer cases from January 2016 have to be registered. Proper predictions using this model would be available in future studies. It is also noteworthy that we did not conduct Nordpred predictions for each secondary medical region due to the uncertain precision. In a previous study, predictions using the APC model were conducted using the data for the whole country, in which the population ranged from 1.4 million to 63.2 million [36, 37], while the population of secondary medical areas ranged from 1.5 million to 350,000, which is usually much smaller than those of whole areas (Table 1, S3 Table). A smaller population would cause a wider variance in the incidence, in addition to the wider range in the preciseness of the cancer registry data for each region.

Third, the number of patients per BC specialist was used as an index of the current medical care system for BC. Two main models, based on the required visits for clinics per person [38] or using the predicted demand estimated by the gross domestic product levels [39], have been used for the estimation of the appropriate number of doctors at the national level. However, it is unclear whether these methods are suitable for estimating medical care for a single condition in a small area (e.g., secondary medical regions). Further studies are warranted and will allow proper interpretation of the present results.

In conclusion, this study demonstrated that the number of BC patients, especially elderly patients, is predicted to increase in urban areas. This study indicated that different types (quality) and supply (quantity) of medical care would be required based on the characteristics of the area, and such differences need to be considered in future cancer care plans. Many developed countries, including Japan, will encounter the problems associated with an aging society, and this study provides extremely useful information on these future issues.

## Supporting Information

S1 Fig Kanagawa Prefecture and the Tokyo Metropolitan Area. This map shows the positional relationship of Kanagawa Prefecture to the Tokyo metropolitan area. The geographical information system (GIS) data of the map was used under a CC BY license, with permission from Mitsui Zosen Systems Research Inc., original copyright 2010.
(TIF)

S2 Fig Breast Cancer Mortality and Incicence Rate for those Aged ≥65 Years in Japan (/100,000 Population). In recent years, the age-specific mortality and incidence rate of elderly continue to increase in Japan. The age-specific mortality rate for those aged ≤65 years increased from 31.7 per 100,000 in 2005 to 36.4 per 100,000 in 2009, and the incidence rate increased from 108.2 to 138.5 per 100,000, respectively, over the same period [12].
(TIF)

S3 Fig Data Collection Method (for Each Medical Region). The geographical information system (GIS) data of the map was used under a CC BY license, with permission from Mitsui Zosen Systems Research Inc., original copyright 2010.
(TIF)

S4 Fig The 11 Secondary Medical Regions in Kanagawa Prefecture. The geographical information system (GIS) data of the map was used under a CC BY license, with permission from Mitsui Zosen Systems Research Inc., original copyright 2010.
(TIF)

S5 Fig Trends and Projections of the Annual BC Incidence (case) using Nordpred in Kanagawa for Each Area. Trends and projections of the BC incidence cases for 4 areas (urban, town, outer city and rural areas) in Kanagwa, Japan.
(TIF)

S1 Method Calculating age specific mortality and incidence rate in Japan. (DOCX)

S1 Table Geographical Features of Secondary Medical Regions. (DOCX)

S2 Table Geographical Features of the Four Areas and the Number of Breast Cancer Specialists (Published by The Japanese Breast Cancer Society in April 2015). (DOCX)

S3 Table Estimated Population for Secondary Medical Regions in Kanagawa (Female). The population prediction data was based on the cohort component method by the National Institute of Population and Social Security Research. We used this population data for each secondary medical region.
(DOCX)

S4 Table Estimated Population for Four Areas in Kanagawa (Female). The population prediction data was based on the cohort component method by the National Institute of Population and Social Security Research. We aggregated this population data by the 4 areas for the regional prediction.
(DOCX)
