# Supplementary figures and images for: Prediction of Female Breast Cancer Incidence among the Aging Society in Kanagawa, Japan
Source: PLoS One. 2016 Aug 17;11(8):e0159913. doi: 10.1371/journal.pone.0159913 (PMC4988816; doi:10.1371/journal.pone.0159913)

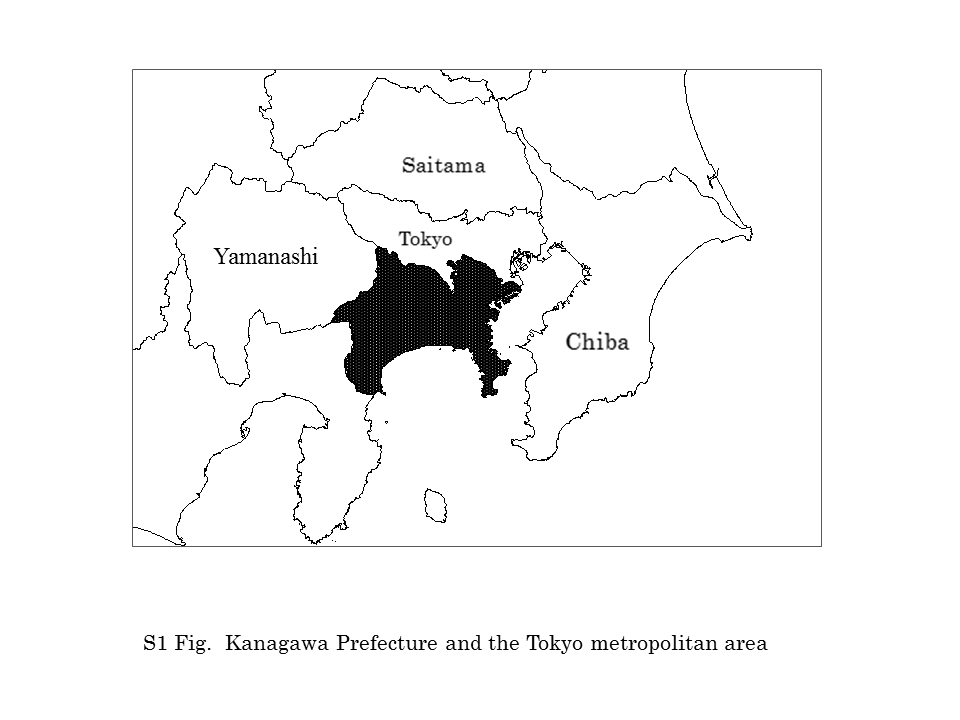

Supplement: S1 Fig — This map shows the positional relationship of Kanagawa Prefecture to the Tokyo metropolitan area. The geographical information system (GIS) data of the map was used under a CC BY license, with permission from Mitsui Zosen Systems Research Inc., original copyright 2010. (TIF) [file pone.0159913.s001.TIF]

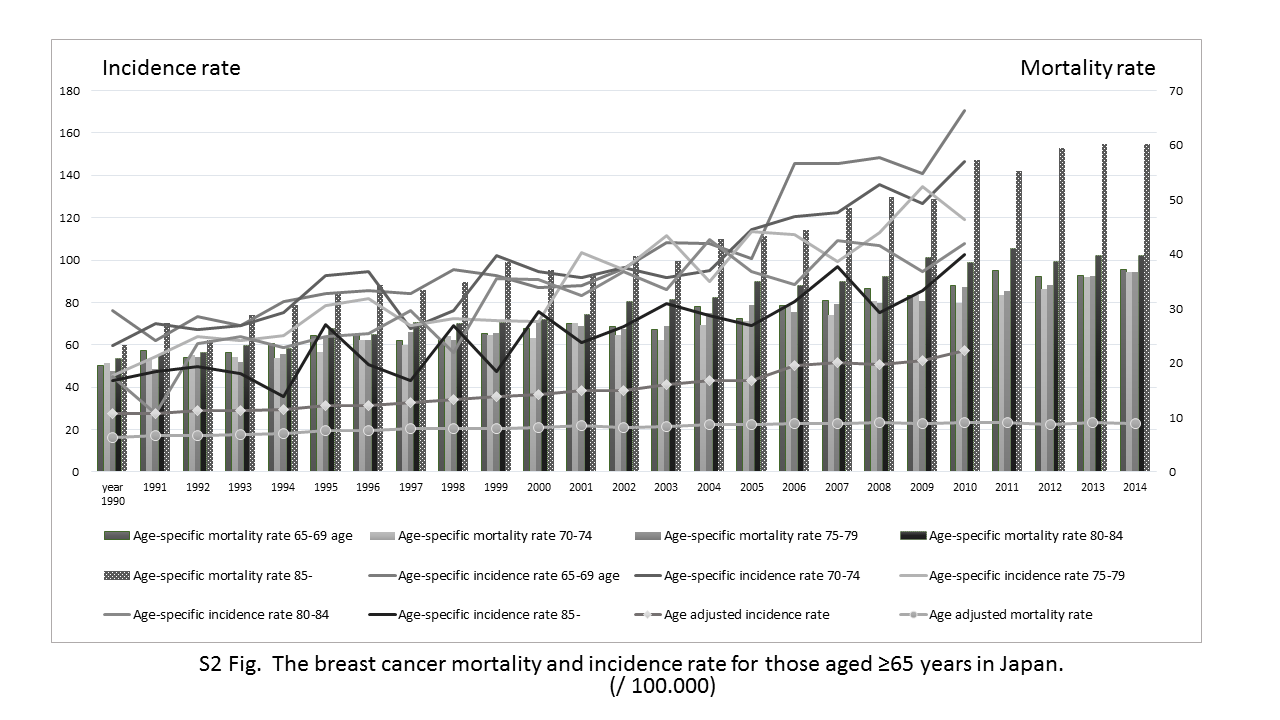

Supplement: S2 Fig — In recent years, the age-specific mortality and incidence rate of elderly continue to increase in Japan. The age-specific mortality rate for those aged ≤65 years increased from 31.7 per 100,000 in 2005 to 36.4 per 100,000 in 2009, and the incidence rate increased from 108.2 to 138.5 per 100,000, respectively, over the same period [12]. (TIF) [file pone.0159913.s002.tif]

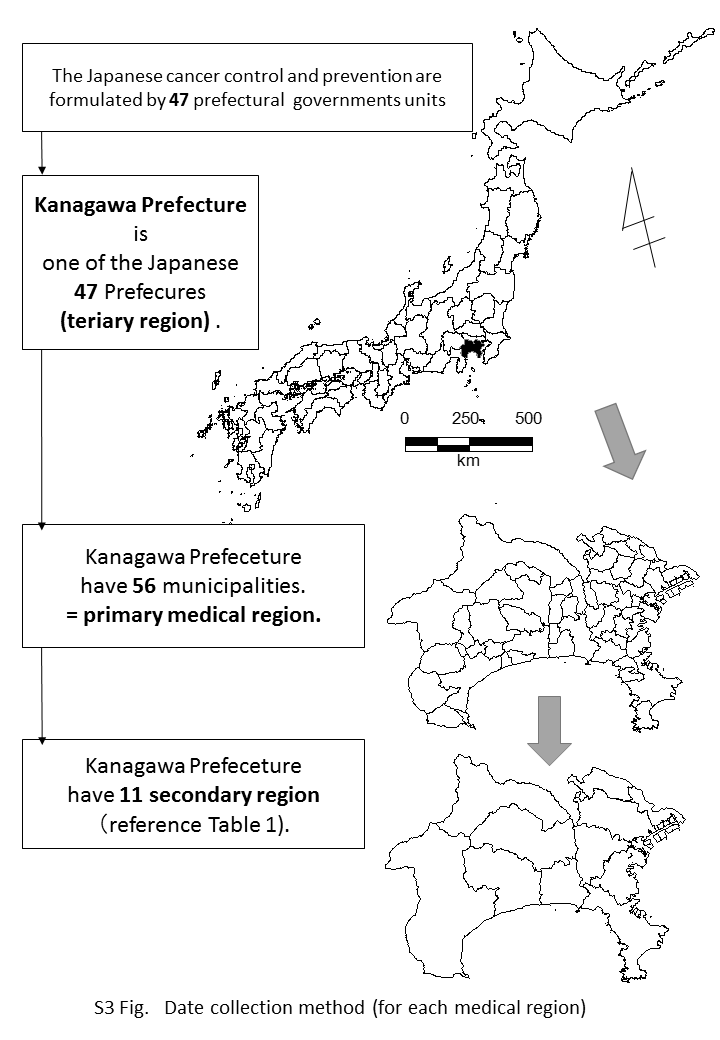

Supplement: S3 Fig — The geographical information system (GIS) data of the map was used under a CC BY license, with permission from Mitsui Zosen Systems Research Inc., original copyright 2010. (TIF) [file pone.0159913.s003.tif]

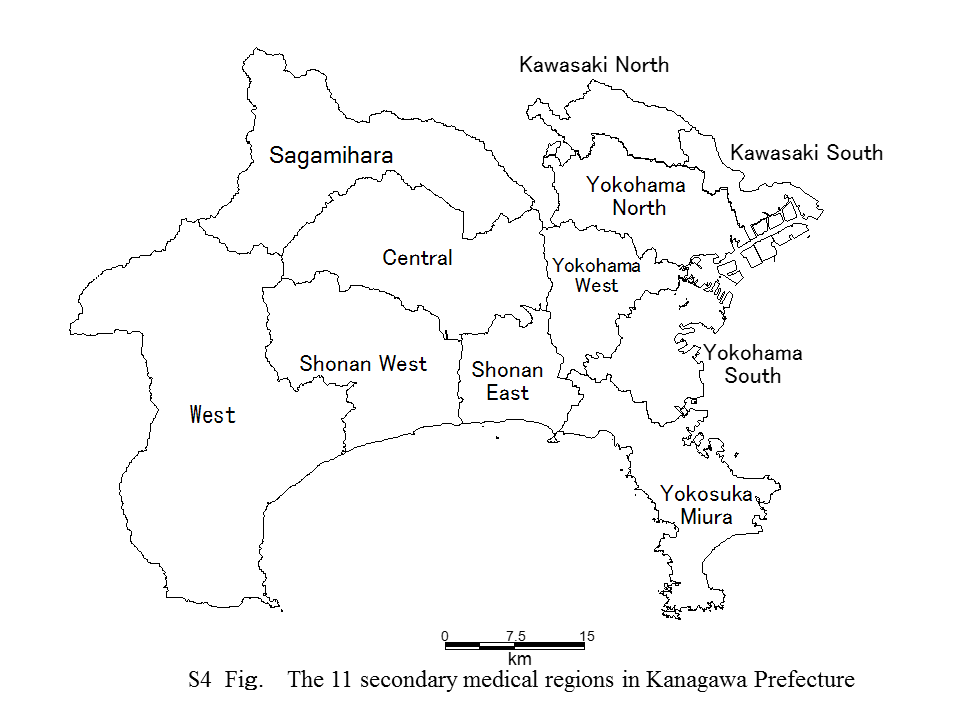

Supplement: S4 Fig — The geographical information system (GIS) data of the map was used under a CC BY license, with permission from Mitsui Zosen Systems Research Inc., original copyright 2010. (TIF) [file pone.0159913.s004.TIF]

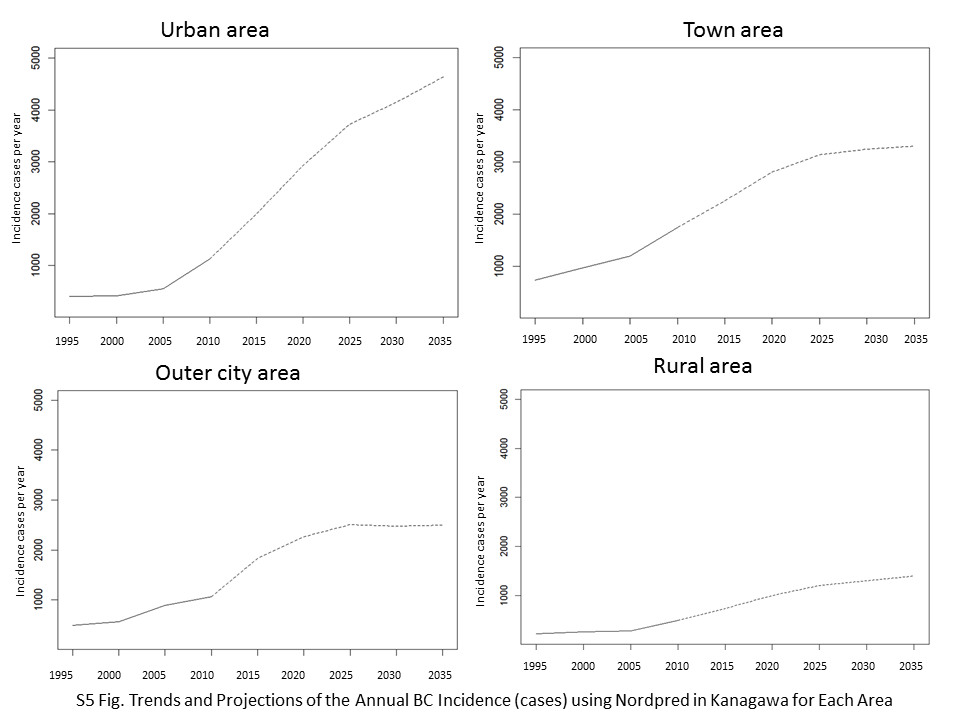

Supplement: S5 Fig — Trends and projections of the BC incidence cases for 4 areas (urban, town, outer city and rural areas) in Kanagwa, Japan. (TIF) [file pone.0159913.s005.TIF]

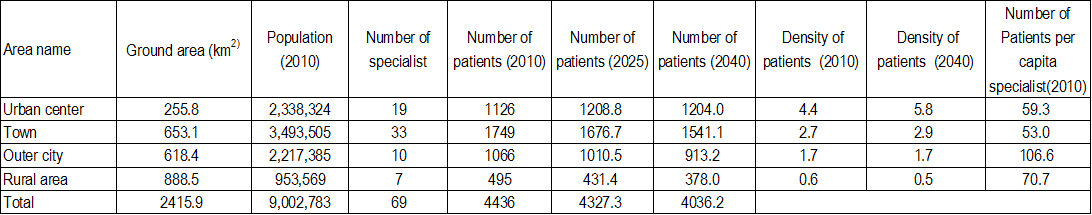

Supplement: S2 Table — (DOCX) [file pone.0159913.s008.docx]
