# Supplementary material for: Prediction of Female Breast Cancer Incidence among the Aging Society in Kanagawa, Japan
Source: PLoS One. 2016 Aug 17;11(8):e0159913. doi: 10.1371/journal.pone.0159913 (PMC4988816; doi:10.1371/journal.pone.0159913)
Supplement: S1 Method — (DOCX) [file pone.0159913.s006.docx]

**S 1 Method. Calculating age specific mortality and incidence rate in Japan**

Using the Vital Statistics Japan data, we used data for 1) breast cancer death cases and 2) female population data from 1990 to 2014. We then calculated age-specific mortality rate among 5-year age groups (aged ≥65 years) and the age-adjusted mortality rate.

The Japan Cancer Surveillance Research Group has been involved in cancer monitoring in Japan since 2000. This group aimed to estimate the cancer incidence in Japan in 2008 based on data collected from 25 of 34 population-based cancer registries, as part of the Cancer Incidence in Japan (MCIJ) project. We used this MCIJ incidence data from 1990 to 2009. Using these data, we calculated the age-specific incidence rate of 5-year age groups (aged ≥65 years) and age-adjusted incidence rate (S2 Fig).
