# Supplementary material for: Prediction of Female Breast Cancer Incidence among the Aging Society in Kanagawa, Japan
Source: PLoS One. 2016 Aug 17;11(8):e0159913. doi: 10.1371/journal.pone.0159913 (PMC4988816; doi:10.1371/journal.pone.0159913)
Supplement: Supplementary file 4 [file pone.0159913.s009.docx]

Secondary medical region	Age group (years)	2010	2015	2020	2025	2030	2035	2040
Yokohama North	0-14	103,989	101,697	96,324	89,608	83,069	79,887	77964
	15-39	249,132	230,495	218,338	210,688	206,677	200,068	188958
	40-64	252,031	269,191	282,454	290,295	283,523	264,370	242962
	≥65	144,321	178,698	198,460	213,518	233,768	260,429	287569
	total	749,473	780,081	795,576	804,109	807,037	804,754	797453
Yokohama West	0-14	71,493	68,056	62,868	56,984	51,664	48,692	46798
	15-39	165,716	147,447	137,026	131,078	127,274	120,889	111923
	40-64	185,846	187,825	189,258	188,900	180,182	164,968	147403
	≥65	135,824	163,439	176,173	181,101	187,179	196,835	208282
	total	558,876	566,767	565,325	558,063	546,299	531,384	514406
Yokohama South	0-14	62,540	59,448	54,414	48,922	43,801	40,670	38451
	15-39	154,934	135,868	124,388	116,550	110,998	104,561	96408
	40-64	182,817	180,426	179,152	176,894	167,531	151,702	133737
	≥65	130,367	155,977	167,212	170,748	174,599	180,677	187684
	total	530,660	531,719	525,166	513,114	496,929	477,610	456280
Kawasaki North	0-14	54,262	54,886	53,148	49,829	46,265	44,399	43095
	15-39	144,774	133,842	125,112	119,490	116,392	112,591	107907
	40-64	133,247	144,725	154,996	162,826	161,983	153,172	140463
	≥65	74,970	94,450	106,128	114,590	126,001	141,114	157251
	total	407,250	427,903	439,384	446,735	450,641	451,276	448716
Kawasaki South	0-14	36,549	37,597	37,204	35,498	33,234	31,472	30136
	15-39	103,626	94,107	87,041	80,058	76,864	74,468	72176
	40-64	90,647	97,100	105,163	113,210	114,528	109,802	99848
	≥65	58,914	66,998	71,109	72,456	75,755	82,422	92358
	total	289,737	295,802	300,517	301,222	300,381	298,164	294518
Yokosuka, Miura	0-14	43,648	40,644	36,462	32,423	29,020	27,011	25720
	15-39	96,498	84,208	78,069	74,787	71,828	67,389	61280
	40-64	125,007	120,067	116,938	113,570	106,455	96,351	84668
	≥65	109,395	123570	127,156	124,768	123,026	122,937	124954
	total	374,548	368,489	358,625	345,548	330,329	313,688	296622
Shonan East	0-14	47,182	46,023	42,654	38,855	35,675	33,932	32895
	15-39	106,284	95,667	89,811	87,174	85,738	82,300	77511
	40-64	117,315	119,612	122,198	123,312	118,609	110,457	99377
	≥65	78,421	94,184	102,051	105,210	109,564	115,626	123641
	total	349,202	355,486	356,714	354,551	349,586	342,315	333424
Shonan West	0-14	37,785	35,377	32,253	29,142	26,441	24,756	23519
	15-39	86,404	77,190	71,366	67,440	64,333	60,047	55010
	40-64	99,942	96,500	94,347	93,169	89,147	83,095	74847
	≥65	69,004	83,878	91,946	94,531	96,378	98,245	100999
	total	293,135	292,945	289,912	284,282	276,299	266,143	254375
Central	0-14	55,685	53,181	49,326	45,033	40,923	38,510	36962
	15-39	128,856	114,559	105,984	100,804	98,107	93,244	86630
	40-64	140,281	139,354	139,771	140,569	135,252	125,009	111877
	≥65	85,919	107,789	119,436	123,573	127,328	133,093	140617
	total	410,742	414,883	414,517	409,979	401,610	389,856	376086
Sagamihara	0-14	45,633	43,594	40,643	37,156	33,708	31,668	30133
	15-39	115,718	103,290	95,319	89,880	85,412	79,282	73266
	40-64	119,768	120,328	121,103	122,117	118,697	111,007	99796
	≥65	75,032	94,322	105,403	110,717	116,269	123,424	131317
	total	356,150	361,534	362,468	359,870	354,086	345,381	334512
West	0-14	22,154	19,994	17,843	15,920	14,290	13,275	12535
	15-39	49,306	43,776	40,330	37,697	35,529	32,692	29292
	40-64	62,124	58,703	56,037	54,121	50,859	46,555	41864
	≥65	50,419	57,526	60,627	60,579	60,068	59,917	60084
	total	184,003	179,999	174,837	168,317	160,746	152,439	143775
Total (Kanagawa female population)		4,503,776	4,575,608	4,583,041	4,545,790	4,473,943	4,373,010	4,250,167
