# Supplementary material for: Prediction of Female Breast Cancer Incidence among the Aging Society in Kanagawa, Japan
Source: PLoS One. 2016 Aug 17;11(8):e0159913. doi: 10.1371/journal.pone.0159913 (PMC4988816; doi:10.1371/journal.pone.0159913)
Supplement: Supplementary file 5 [file pone.0159913.s010.docx]

Area name	age group (years)	2010	2015	2020	2025	2030	2035	2040
Urban	0-14 years	158,251	156,583	149,472	139,437	129,334	124,286	121059
	15-39	393,906	364,337	343,450	330,178	323,069	312,659	296865
	40-64	385,278	413,916	437,450	453,121	445,506	417,542	383425
	≥65	219,291	273,148	304,588	328,108	359,769	401,543	444820
	total	1,145,351	1,207,984	1,234,960	1,250,844	1,257,678	1,256,030	1,246,169
Town	0-14 years	216,215	208,695	195,129	178,560	162,407	152,502	145518
	15-39	539,994	480,712	443,774	417,566	400,548	379,200	353773
	40-64	579,078	585,679	594,676	601,121	580,938	537,479	480784
	≥65	400,137	480,736	519,897	535,022	553,802	583,358	619641
	total	1,735,423	1,755,822	1,753,476	1,732,269	1,697,695	1,652,539	1,599,716
Outer city	0-14 years	146,515	139,848	128,442	116,311	105,618	99,453	95577
	15-39	331,638	294,434	273,864	262,765	255,673	242,933	225421
	40-64	382,603	379,033	378,907	377,451	360,316	331,817	295922
	≥65	273,735	325,543	348,643	353,551	359,918	371,656	389212
	total	1,134,492	1,138,858	1,129,856	1,110,078	1,081,525	1,045,859	1,006,132
Rural area	0-14 years	59,939	55,371	50,096	45,062	40,731	38,031	36054
	15-39	135,710	120,966	111,696	105,137	99,862	92,739	84302
	40-64	162,066	155,203	150,384	147,290	140,006	129,650	116711
	≥65	119,423	141,404	152,573	155,110	156,446	158,162	161083
	total	477,138	472,944	464,749	452,599	437,045	418,582	398,150
Total (Kanagawa female population)		4,492,404	4,575,608	4,583,041	4,545,790	4,473,943	4,373,010	4,250,167
